# An Enhanced Social Network Strategy to Increase the Uptake of HIV Services: Protocol for Type I Hybrid Implementation Study (Carolinas RESPOND)

**DOI:** 10.2196/69495

**Published:** 2025-08-29

**Authors:** Ian Dale, Meagan Zarwell, Alicia Diggs, Jesse Strunk Elkins, Eunice Okumu, Sharon Weissman, Feng-Chang Lin, Victoria Mobley, Carol Golin, Abby Rudolph, Ann M Dennis

**Affiliations:** 1 Institute for Global Health and Infectious Diseases, University of North Carolina at Chapel Hill, Chapel Hill, NC, United States; 2 Department of Epidemiology and Community Health, College of Health and Human Services, University of North Carolina at Charlotte, Charlotte, NC, United States; 3 Center for AIDS Research, University of North Carolina at Chapel Hill, Chapel Hill, NC, United States; 4 School of Medicine, University of South Carolina, Columbia, SC, United States; 5 Gillings School of Global Public Health, University of North Carolina at Chapel Hill, Chapel Hill, NC, United States; 6 Division of Public Health, Communicable Disease Branch, NC Department of Health and Human Services, Raleigh, NC, United States; 7 College of Public Health, Temple University, Philadelphia, PA, United States; 8 School of Medicine, University of North Carolina at Chapel Hill, 130 Mason Farm Rd, Chapel Hill, NC, 27599-6134, United States, 1 9199662536

**Keywords:** social networks, HIV-1, public health surveillance, clinical research protocol, implementation science

## Abstract

**Background:**

In the United States, persisting new HIV diagnoses among gay, bisexual, and other cisgender men who have sex with men (GBMSM) and transgender women make it unlikely that the United States will meet the Ending the HIV Epidemic’s (EHE) goal to reduce new HIV diagnoses by 90% by 2030. Innovative strategies are needed to address this challenge, particularly in the US South, where Black and Latinx GBMSM and transgender women are disproportionately impacted by HIV. Social network approaches have led to increased HIV testing uptake. Social network interventions that are responsive to individuals’ needs among disproportionately impacted groups could also increase engagement across the HIV prevention and care continuum.

**Objective:**

This hybrid type 1 effectiveness-implementation study will evaluate an enhanced social network strategy (eSNS) intervention designed to increase engagement in HIV services (HIV testing, pre-exposure prophylaxis [PrEP] use, and HIV care) by groups disproportionately affected by HIV. From 2025 to 2027, eSNS will be delivered in the Charlotte, North Carolina (NC) region, which includes Mecklenburg County, a priority EHE jurisdiction.

**Methods:**

The study’s phase 1 was a formative period of mixed methods data collection to operationalize enhancements to the Centers for Disease Control and Prevention’s social network strategy (SNS). In Phase 2, the intervention will be integrated into standard NC Partner Services for people diagnosed with HIV and their sexual or social contacts, which is routinely performed by disease intervention specialists (DISs). We will identify network recruiters (ambassadors) who are 18 years and older and are either reached by study team DIS (DIS coaches) performing partner services or referred at community sites. Over 2‐6 weeks, DIS coaches will guide ambassadors to identify and refer people in their network (peers) for HIV services and will facilitate peers’ referrals to HIV services. Finally, Phase 3 will evaluate the eSNS’s effectiveness in increasing HIV services uptake compared to standard-of-care partner services in the Raleigh, NC region.

**Results:**

This project was funded by the National Institutes of Health and initially approved by the University of North Carolina at Chapel Hill’s Institutional Review Board in 2022. Phase 1 concluded in August 2024. Implementation of eSNS (Phase 2) was launched in March 2025. Based on phase 1 findings, the study was modified to include Ambassadors of any race or ethnicity and gender (originally only Black GBMSM and transgender women) and expand identification of ambassadors through community sites (in addition to partner services).

**Conclusions:**

Substantial reductions in new HIV diagnoses depend on public health approaches that effectively reach people with a higher likelihood of acquiring HIV. Our protocol proposes integrating existing strategies with an innovative intervention (eSNS) to reduce social barriers to disproportionately affected groups’ engagement in the full HIV prevention and care continuum.

## Introduction

Innovative public health strategies are needed to increase engagement in HIV prevention and care services in the United States, and thereby curb onward HIV transmission. Gay, bisexual, and other cisgender men who have sex with men (GBMSM) and transgender women, particularly Black and Latin individuals residing in the US South, are disproportionately impacted by HIV due to structural barriers to health care and their position in networks with a higher prevalence of HIV [1]. In 2022, 52% of all HIV diagnoses in the United States occurred in the South; of those, 47% were people who identify as Black or African American and 26% among people who identify as Hispanic or Latino, despite representing only 19% and 18% of the region’s population, respectively [2]. Cultural and structural barriers such as stigma, medical mistrust, and geographic distance further hinder engagement with care and prevention services for Black and Latinx GBMSM and transgender women [3-5].

Partner services is a longstanding strategy conducted by some local and state health departments to reduce HIV transmission, usually by deploying disease intervention specialists (DISs) to offer testing and linkage to care or prevention services to sex or injecting partners of people newly diagnosed or reported with HIV [6]. While there is evidence that partner services yield a relatively high proportion of new HIV diagnoses among identified partners [7-9], including when compared to interventions where people newly diagnosed with HIV notify partners themselves [10], reaching sex or injecting partners through partner services can be challenging. Historic increases in DIS caseloads and clients unable to recall information about sex partners met on dating apps have reduced the feasibility of partner notification [11], while health departments may not always be able to interview people with new HIV diagnoses to request partners’ names or conduct in-person field investigations [12], which have better partner outcomes compared to telephone-based partner services [13]. In addition, partner services frequently fail to identify sources of infection (ie, transmitting partner) [14-16]. Given the disproportionate and concentrated burden of HIV among Black and Latinx GBMSM and transgender women, there is an urgent need for interventions that better meet these populations’ specific needs.

Strategies that leverage existing social connections, thereby reducing stigma and increasing trust, may be highly impactful among GBMSM and transgender women, where sexual networks that are disproportionately burdened by HIV are also frequently homogeneous by race and gender [17]. The Centers for Disease Control and Prevention (CDC)’s social network strategy (SNS) has been effective at increasing HIV testing among populations disproportionately affected by HIV [18-22]. The SNS program coaches people from high-incidence social or sexual networks to recruit their peers (eg, friends, not only sex or injecting partners) to engage in HIV testing. By leveraging trust among members of these social networks, SNS operates on the principle that people are more likely to respond positively to health behaviors and messages about engaging in HIV services from people in their social circle rather than unfamiliar people, such as DIS or other public health workers. By supplementing existing partner services programs with SNS, we hypothesize that a combined partner services-SNS intervention package could reach more individuals needing HIV services by implementing it among social networks with recent HIV transmission. A focus on networks involving recent HIV transmission (ie, acute or early HIV) may be optimal for promoting early antiretroviral therapy (ART) and uptake of pre-exposure prophylaxis (PrEP) among network members, thereby reducing further transmission. In North Carolina, individuals diagnosed with acute HIV infection reported having more sexual partners than those diagnosed with chronic HIV, providing an opportunity to intervene in networks with high probability of HIV acquisition [23]. Combining contact network and HIV genotype data with social network strategies can further enhance our ability to identify and intervene in networks whose members have a higher probability of acquiring HIV, potentially preventing onward HIV transmission [14,15]. Molecular epidemiology uses HIV sequences derived from genotypes to identify clusters of highly similar sequences that indicate recent transmission [24,25]. An approach that integrates SNS, standard partner services, and molecular epidemiology may provide a “next generation” public health strategy that better connects disproportionately affected networks with HIV prevention and care services.

In this paper, we present the protocol for such an integrated approach: an enhanced social network strategy (eSNS) intervention, which is part of the Carolinas RESPOND study. Adapted from CDC’s SNS, our eSNS enhances SNS in three key ways: (1) Focusing on engagement in PrEP and HIV care for social network members, not only on HIV testing, (2) integration with partner services, and (3) expanding potential networks to include HIV genetic cluster members identified by molecular epidemiology. From 2025 to 2027, we will implement eSNS in the Charlotte region of North Carolina, which includes Mecklenburg County and directly borders the state of South Carolina; both are regions prioritized by the Ending the HIV Epidemic (EHE) initiative [26]. Our eSNS will prioritize networks involving recent HIV transmission, which we anticipate will most frequently be comprised of Black and Latinx GBMSM and transgender women but may include participants of any race or gender (including nonbinary individuals) [27]. The intervention will be delivered alongside standard partner services to better direct eSNS toward networks with recent HIV diagnoses, particularly those with acute or recent HIV infection and clusters with rapid HIV transmission. The study will evaluate the effectiveness of eSNS to (1) increase HIV prevention and treatment uptake among these key populations and (2) identify HIV transmission networks connected via sexual or injecting relationships or characterized by closely related viral infections and ongoing HIV transmission. This protocol describes our formative data collection efforts, intervention procedures, evaluation framework, and key adaptations.

## Methods

### Study Aims

The Carolinas RESPOND study’s aims are to: (1) assess how genetic sequence analysis added to standard surveillance impacts spatial and demographic features of HIV networks in juxtaposed high burden regions of North Carolina and South Carolina; (2) identify barriers and facilitators to engagement in HIV prevention and treatment services by individuals from communities disproportionately impacted by HIV who are part of networks with HIV recent transmission; and (3) evaluate an intensified partner services response using eSNS, which leverages HIV genetic cluster analysis-based network characterization. Applying genetic sequence analyses used in cluster detection and response will provide an additional tool to direct eSNS within networks experiencing recent HIV transmission. This protocol describes the plans for eSNS adaptation and implementation in the RESPOND study.

### Study Setting

**Figure 1.**
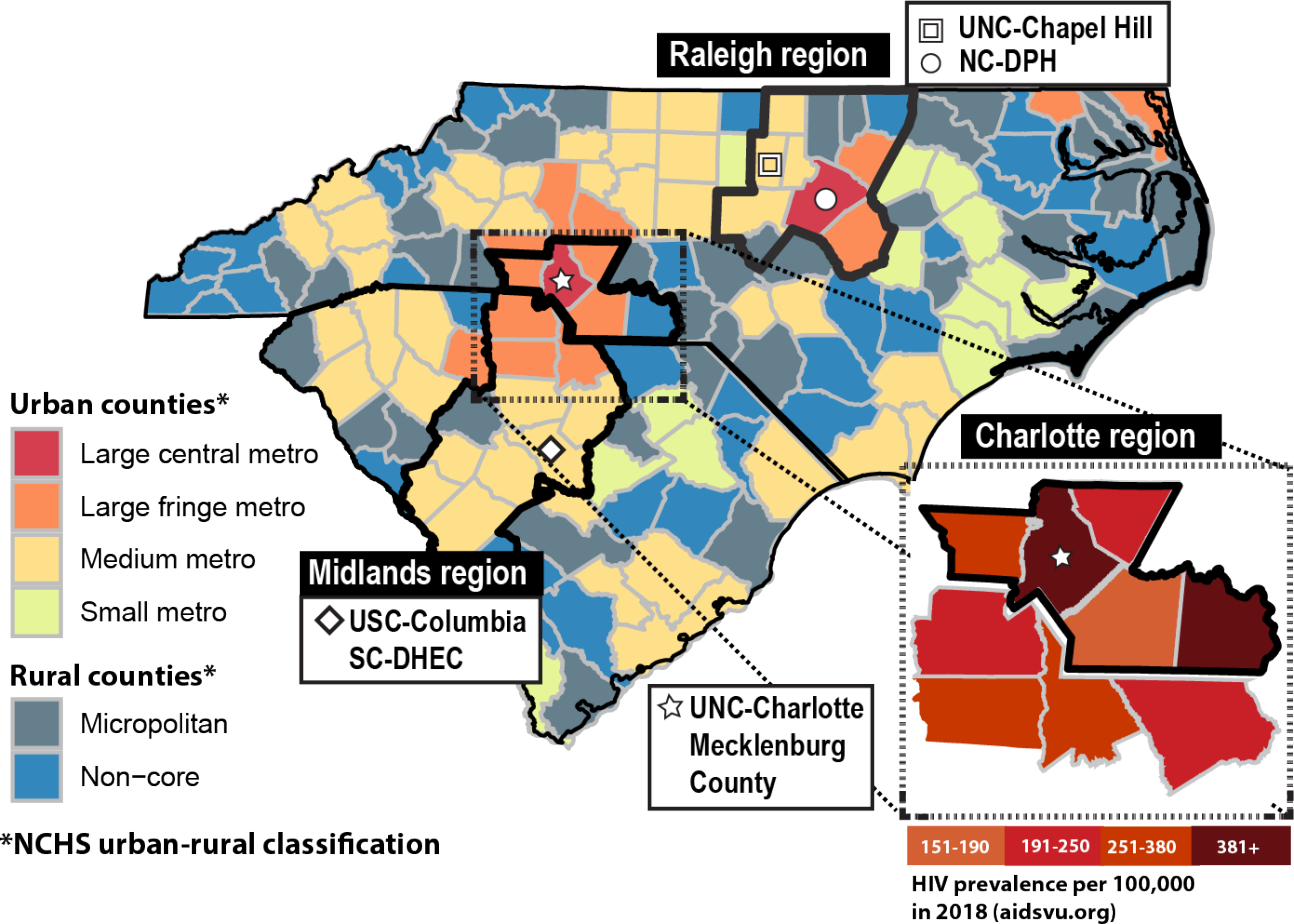
Map of study setting. The enhanced social network strategy (eSNS) will be implemented in the 5-county area of North Carolina Department of Health and Human Services (DHHS) Field Services Region II (Charlotte region shown bordered in black in the inset at the right). North Carolina DHHS Field Services Region IV (Raleigh, 11 counties) is shown bordered. UNC: University of North Carolina; NC-DPH: North Carolina Division of Public Health; USC: University of South Carolina; SC-DHEC: South Carolina Department of Health and Environmental Control.

The Carolinas RESPOND study focuses on North Carolina and South Carolina, with collaboration among academic and public health partners in both states (see Figure 1). The eSNS intervention described in this protocol is planned for the Charlotte region, covering a 5-county area in the North Carolina Department and Health and Human Services’ (DHHS) Field Services Region II, which includes the EHE priority jurisdiction [28] of Mecklenburg County (with star in Figure 1). This region directly borders the Midlands region of South Carolina, one of 7 US states with a disproportionate burden of HIV in rural areas [26]; the regions are connected by Interstate 77, which runs from Charlotte, North Carolina, to Columbia, South Carolina (see diamond in Figure 1). Although implementation is focused on the Charlotte region, our eSNS will follow networks that may cross both states because of Charlotte’s geospatial proximity to South Carolina. Due to comparable demographics and HIV epidemiology [27], North Carolina DHHS’s Field Services Region IV, the 11-county area surrounding the Raleigh, North Carolina metropolitan area, was chosen as a “control” region against which outcomes in the Charlotte, North Carolina “intervention” region will be compared.

### Theoretical Framework

Our study design is guided by the modified social ecological model (MSEM) [29]. This model examines the likelihood of HIV acquisition through multiple levels by situating individual behaviors within network, community, sociostructural, and epidemic contexts. Depending on network structures and underlying prevalence, individual behaviors may either facilitate or hinder HIV acquisition. Understanding behaviors that may protect one from or increase one’s chances of acquiring HIV within the context of individuals’ connections can inform more effective strategies to prevent ongoing transmission [29]. Within the MSEM model, we draw on the theory of how social networks impact health [30], wherein social networks are mezzo-level features that are shaped by macro-level social structural conditions and capable of influencing micro-level social and interpersonal behaviors. For example, individuals who are connected by sex or injecting partnerships within social networks characterized by a high HIV prevalence (or incidence), or who bridge to other networks by having sex or injecting partnerships with 1 or more individuals in distinct networks, may have an increased likelihood of acquiring HIV. At the behavioral level, networks can also have positive influences, such as the provision of social support, informational support, emotional support, social engagement and attachment, and access to resources and material goods. Just as HIV can be transmitted across networks, health information and behavior changes can similarly diffuse through networks [31,32]. Interventions that leverage existing networks of relationships can promote healthier behavior norms through social influence (eg, increased HIV testing and PrEP uptake) [33].

### Study Design

**Figure 2.**
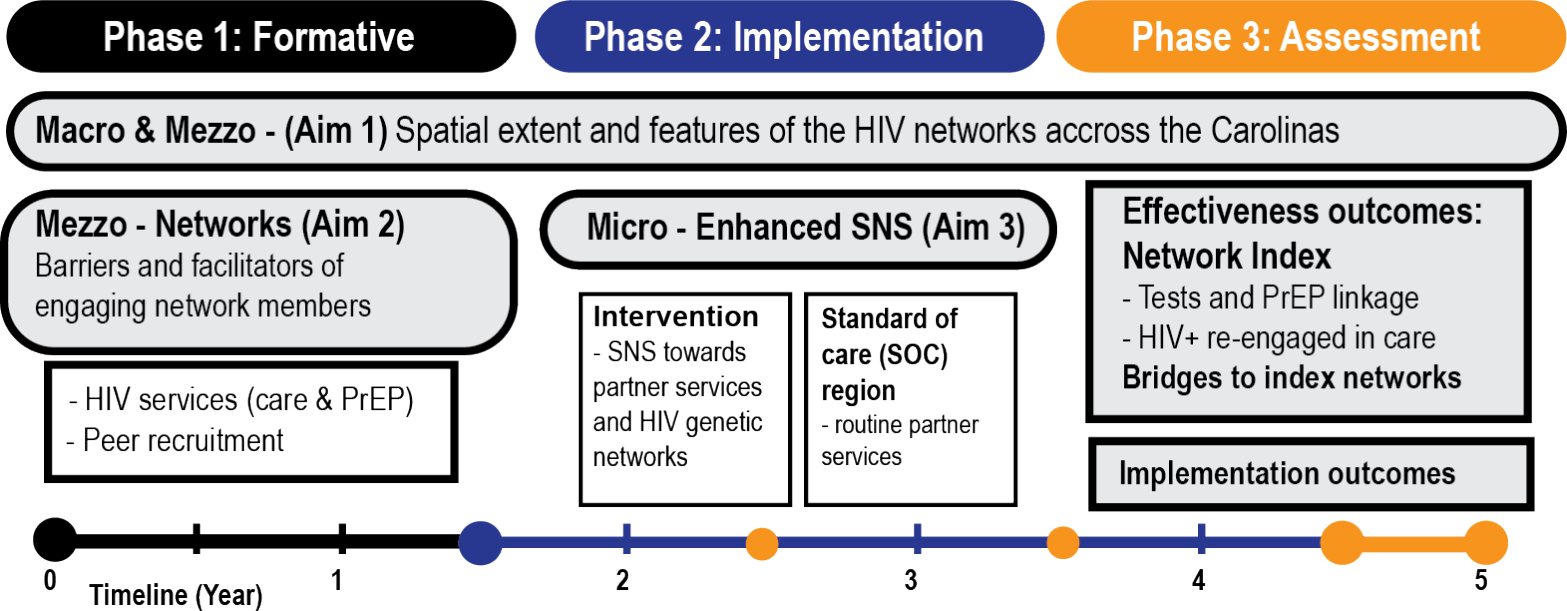
Schematic overview of the study design. SNS: social network strategy; PrEP: pre-exposure prophylaxis.

We designed a 3-phase process (see Figure 2) to develop, implement, and evaluate eSNS using a quasi-experimental, nonrandomized hybrid type 1 effectiveness-implementation design [34]. This design allows for evaluation of both eSNS effectiveness and the intervention delivery process. Phase 1 (preimplementation), which involved establishing a community advisory group and collecting mixed methods data to inform adaptations to SNS procedures, concluded in August 2024. Analyses of Phase 1 data, which are ongoing, aim to understand the structural, spatial, and network factors that influence HIV services engagement among the study’s priority groups, plus barriers and facilitators to eSNS implementation. In Phase 2, eSNS will be implemented in the Charlotte region over a 3-year period (2025‐2027). In Phase 3, we will evaluate the impact of eSNS on HIV services use compared to standard-of-care partner services delivered in the Raleigh, North Carolina metropolitan region [27] (see Figure 1).

### Implementation Research Logic Model

**Figure 3.**
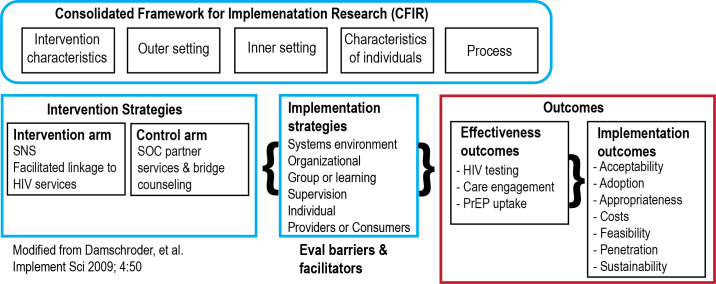
Conceptual implementation science framework for evaluating enhanced social network strategy (eSNS). PrEP: pre-exposure prophylaxis.

We use the Consolidated Framework for Implementation Research (CFIR) [35] to guide exploration of potential barriers and facilitators to eSNS implementation and to inform a strategy for regional scale-up and sustainability (see Figure 3). The CFIR offers a comprehensive and widely used framework for categorizing potential determinants of intervention implementation. We chose a hybrid type 1 design because it has the advantage of speeding translation of eSNS toward successful implementation by assessing implementation barriers and effectiveness simultaneously [34]. Integrating this design with the CFIR demonstrates where implementation determinants will be assessed in implementation planning.

### Community Advisory Group

A community advisory group (CAG) led by a co-investigator (AD) with lived experience was first formed in August 2023. Members were initially invited to join from the Charlotte-area researchers’ professional networks, community organizations and clinics, and community initiatives. The group meets quarterly, and attendees are provided an honorarium of US $100 after each meeting. We plan to maintain a roster of approximately 10 CAG members at any given time; during phase 2, interested eSNS participants will also be invited to join. Our CAG will be consulted throughout the study to share local resources, to advise about appropriate community engagement strategies and study participant compensation, and to provide feedback on the study’s recruitment materials, participant engagement language, and social media presence.

### eSNS Intervention Overview

**Figure 4.**
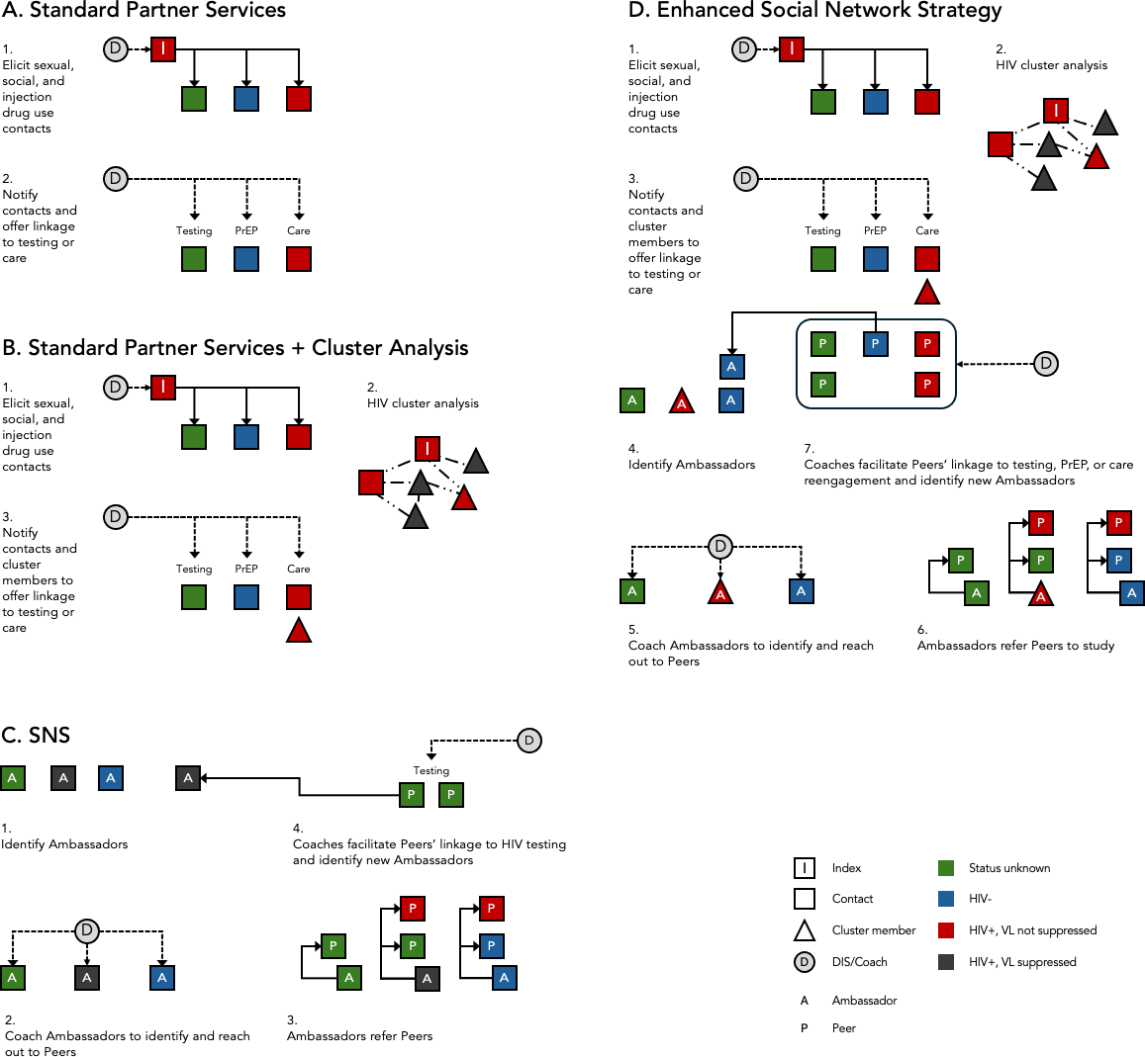
Schematic overview of enhanced social network strategy (eSNS) intervention among priority networks identified during routine partner services. A. Partner services as routinely delivered by disease intervention specialists (DIS) eliciting contacts (usually sexual or injecting partners) for notification of exposure to HIV. B. Partner services when combined with HIV cluster response, where genetic cluster members who are not virally suppressed are also linked to care services. C. Standard social network strategy (SNS), where potential network recruiters (ambassadors) are identified from a population of interest, then coached to reach out to network members (peers) for HIV testing. D. eSNS, which combines all 3 strategies. SOC: standard of care; PrEP: pre-exposure prophylaxis.

The eSNS intervention will be implemented by DIS coaches toward priority networks, which are networks with recent HIV transmission involving key population members (including Black or African American, Latin GBMSM, and transgender women). DIS coaches are study team members responsible for delivering both the eSNS intervention and routine partner services (see Textbox 1). Priority networks will be identified through routine reporting of positive HIV tests to the North Carolina DHHS HIV surveillance system, which are then assigned for partner services by state and county DIS. The eSNS intervention will initially identify priority network members (index persons) by assigning DIS coaches to reach individuals aged 13 years and older (classified by the CDC as adults for HIV surveillance [36]). DIS coaches will be preferentially assigned to index persons diagnosed with acute HIV infection or less than 30 years old with chronic (antibody-positive) HIV, as younger persons are more likely to have recent HIV infection, but will also provide partner services to index persons who are virally unsuppressed and identified as part of an HIV transmission cluster (cluster member; see Figure 4).

Textbox 1. **Definitions of study population and key roles within the enhanced social network strategy (eSNS).**

1. Key population:
  - Member of a group disproportionately affected by HIV (eg, Black or African American or Latine gay, bisexual, and other cisgender men who have sex with men or transgender women).
2. Priority network:
  - Sexual (or injecting) network with ≥1 new HIV diagnosis within 12 months.
  - Identified through reporting of new HIV diagnosis.
3. Community ambassador:
  - Key population member or priority network member.
  - Aged 18 years and older.
  - Resides in the Charlotte region.
  - Able to provide informed consent in English and willing to refer peers to HIV services.
4. Peer:
  - Person in the social, sexual, or injecting network of the ambassador, whom the ambassador believes could benefit from HIV services.
  - Aged 18 years and older.
  - Resides in the Charlotte region or nearby such that they can attend an HIV service appointment in the Charlotte region.
5. Disease intervention specialist (DIS) coach:
  - Study team member who conducts routine partner services for members of priority networks.
  - Also delivers eSNS intervention.

Following the completion of routine partner services activities (contact tracing and voluntary partner notification), potential community ambassadors will be identified from among members of Priority Networks reached through partner services, including index persons and their social and sexual contacts (see Figure 4). DIS coaches will then lead ambassadors through a semistructured coaching session designed to develop an action plan for ambassadors to reach out to and refer their peers—who may be friends or acquaintances, not necessarily sexual or injecting partners—for HIV prevention and care services. Once peers contact the study team, DIS coaches will assist peers in accessing HIV testing, care, or PrEP services.

#### Overview of Key Roles

DIS coaches are trained study staff responsible for delivering eSNS with the support of the study coordinator in Charlotte. These staff members have dual roles as public health DIS and coaches for community ambassadors. As DIS, they receive training and credentials from NC DHHS to perform routine partner services: HIV or sexually transmitted infection (STI) notification, counseling, testing, and linkage to care services for persons with new HIV diagnoses and their social and sexual partners. As coaches, they will identify, enroll, and coach community ambassadors for eSNS. DIS coaches will provide peers with referrals to HIV prevention and treatment services and assist with scheduling appointments.

Community ambassadors are individuals from the study’s key population, who could be referred to eSNS as an index person recently diagnosed with HIV; as a priority network member, that is, a social, sexual, or viral genetic cluster contact of an index person engaged through partner services; or through local community-based organizations (CBOs). While an optimal ambassador will have a confirmed link to a recent HIV transmission network, whether through routine contact tracing or genetic cluster analysis, less emphasis is placed on recruiting the index person as an ambassador because a person with a new HIV diagnosis may be experiencing increased social or psychological stress [37]. After modifying inclusion criteria following Phase 1’s formative findings, eligible ambassadors will include residents of the Charlotte region who are at least 18 years old, report ever having sex with another person assigned male sex at birth, can provide informed consent in English, and indicate their willingness to refer people in their social networks (peers) who they believe may benefit from HIV prevention or treatment services (see Textbox 1).

Peers are people in the social circle of ambassadors (network members) who may be referred to eSNS. Community ambassadors may refer anyone 18 years and older in their social circle whom they believe could benefit from or has reason to use HIV prevention or treatment services. There are no demographic restrictions (aside from age) on who may qualify as a peer, if identified and referred by an enrolled community ambassador. Peers of any sex, gender, or race can be referred from a community ambassador’s social circles if the ambassador reasonably believes they could benefit from HIV prevention or treatment services and the peer lives close enough to attend HIV services in the Charlotte region.

### Phase 1: Evaluate Barriers and Facilitators to Engaging Network Members

For formative data collected during phase 1 (2022‐2024), we used a mixed methods approach to determine the optimal approach to engage members of priority networks and the study’s key population. We collected qualitative data from 2 participant pools, HIV service providers and community members from our key population, to explore local barriers and facilitators to HIV services engagement and general perceptions of eSNS. Between December 2022 and February 2023, we held 4 focus groups and conducted 12 in-depth interviews (IDIs) with Charlotte-area HIV care and prevention services providers (clinicians, public health officials, community organization leaders, HIV case managers, and public health DIS). From July 2023 to February 2024, we conducted in-depth interviews with 13 Black cisgender men who have sex with men and 4 Black transgender women who reported engaging with HIV-related services in the previous 12 months. A total of 13 participants were living with HIV, 3 of whom were diagnosed within 12 months of being interviewed. From April to June 2024, we conducted additional follow-up IDIs with 8 of the 17 participants to further explore their perspectives of barriers and facilitators to implementing specific eSNS components. All IDIs and focus group guides probed aspects of eSNS adaptation and implementation. Interview questions about eSNS implementation were mapped to specific CFIR constructs [38] to maintain consistency across qualitative analyses.

To characterize the social and sexual networks of our key population, including members of priority networks, we created a web-based, self-administered Personal Network Survey (PNS) hosted in REDCap (Research Electronic Data Capture; Vanderbilt University), which we launched in May 2023. A Spanish version was offered beginning in January 2024. Phase 1 survey participants were either referred by a DIS following routine partner services or referred from community organizations and clinics providing HIV services and self-reported engaging in at least one HIV-related service (eg, testing) in the previous 12 months; respondents received a US $20 gift card. The PNS (initial sample n=73, through February 2025) will continue during Phase 2. The PNS was adapted from a quantitative social network assessment survey [39] originally based on the General Social Survey [40]. A total of 3 name generators prompt participants to list pseudonyms of up to 5 individuals (alters) who, in the previous 6 months, provided social support, were sexual partners, or with whom participants used or injected drugs. Respondents can indicate if alters belong to multiple categories. A maximum of 15 unique names is possible. Following the initial inventory, participants are then asked to describe each alter’s demographic characteristics, further categorize their relationship type (“partner,” “family,” “friend,” “advisor or mentor,” “co-worker,” or “other”), indicate the level of closeness with each alter (“especially close” or “not especially close”), and estimate the closeness between alters listed (“stranger,” “in-between,” “especially close,” or “don’t know”). The PNS has been adapted for Phase 2 to include more granular data on social factors that could influence transmission risk, including drug use and chemsex.

### Phase 2: Enhanced Social Network Strategy (ESNS) Intervention

**Figure 5.**
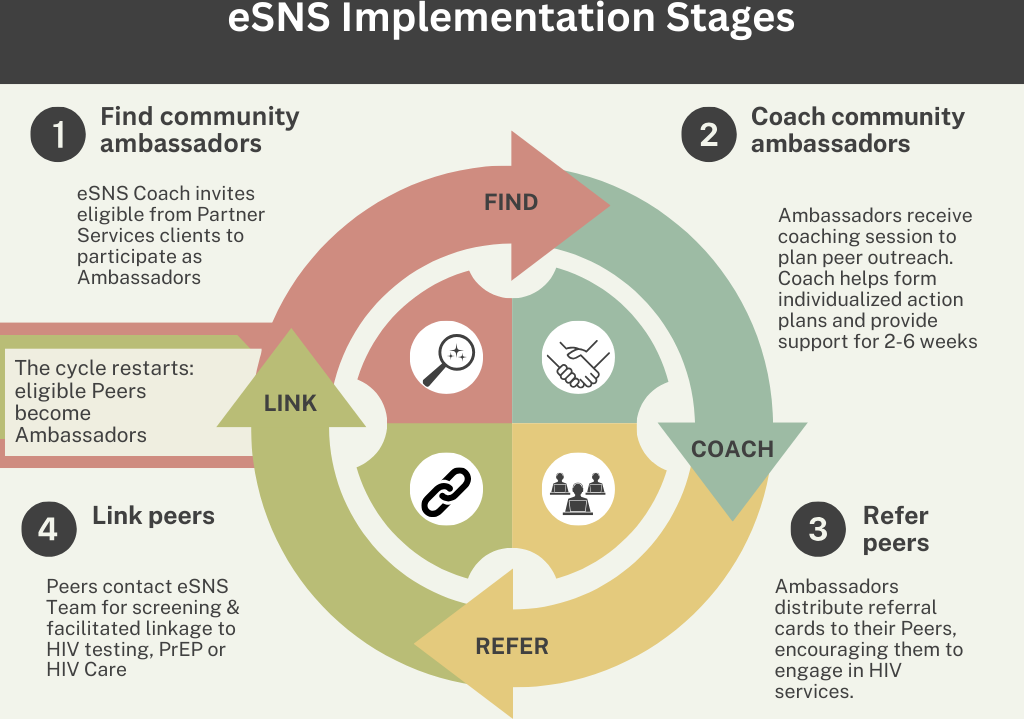
Schematic diagram of enhanced social network strategy (eSNS) procedures, displaying the 4-step intervention structure and revised terminology. SNS: social network strategy; DIS: disease intervention specialist; PrEP: pre-exposure prophylaxis.

In adapting the CDC’s SNS, we retained the original intervention’s 4-step model while renaming the steps with revised terminology that is more person-forward [41] and better meets the needs of our key population (see Figure 5). We will make several adaptations to SNS. Before phase 1, we had planned for eSNS service linkage to include PrEP linkage or care re-engagement (not only HIV testing, unlike SNS) and to embed eSNS within a public health department’s partner services program. Following phase 1, we also widened our key population to include English and non–English-speaking groups disproportionately affected by HIV in North Carolina (although all ambassadors must be able to provide informed consent in English), and, guided by formative data and recommendations of CAG members, developed a process for referrals from community sites to engage key population members outside of partner services (described further in the Results section).

#### Step 1: Finding Community Ambassadors

We will use 3 strategies to enroll a planned sample of at least 20 community ambassadors per year: (1) invite eligible priority network members identified during routine partner services, (2) publicize the study with local community organizations and clinics who provide HIV-related services to the study’s key population, and (3) invite eligible peers to become ambassadors.

For (1), DIS coaches will inform individuals contacted during partner services investigation of priority networks (recent HIV transmission networks involving our key population) about eSNS. To reach networks with the highest probability of recent HIV transmission [42], we will collaborate with NC DHHS to prioritize index persons diagnosed with acute incident HIV infection [43] for assignment to DIS coaches. Following completion of partner services activities, DIS coaches will invite interested clients who meet inclusion criteria to become ambassadors. Eligible clients include people recently diagnosed with HIV, named partners of people recently diagnosed with HIV, or genetic cluster members re-engaged with HIV care who were identified through cluster detection protocols. Ambassadors do not have to be living with HIV.

Based on recommendations from phase 1, for (2) we will identify potential ambassadors through community-based recruitment in addition to partner services. Through partnerships with local CBOs that offer HIV-related services to our key population, we will post print materials at clinic and community venues and distribute digital recruitment materials through social media platforms. For (3), previously referred peers may become ambassadors, facilitating deeper penetration into networks and recruitment of new peers who have not previously been reached. Eligible peers will be contacted by a DIS coach for a brief program orientation before providing informed consent to serve as ambassadors. Newly identified ambassadors will replenish ambassadors who completed peer outreach (Step 3).

#### Step 2: Ambassador Coaching

Following orientation and informed consent, DIS coaches will deliver a semistructured coaching session to ambassadors. Coaching is a core component of eSNS designed to foster rapport and to develop an individualized action plan for contacting peers. We adapted CDC’s SNS Coaching Guide [44] to accommodate findings from Phase 1 formative data and integrated our guide into Network Canvas (Complex Data Collective), an open-source software interface using intuitive visuals to collect personal network data [45]. DIS coaches are encouraged (but not required) to hold the first coaching session in person. All coaching sessions must take place in a private location. Ambassadors will receive a US $35 gift card for completing the coaching session.

**Figure 6.**
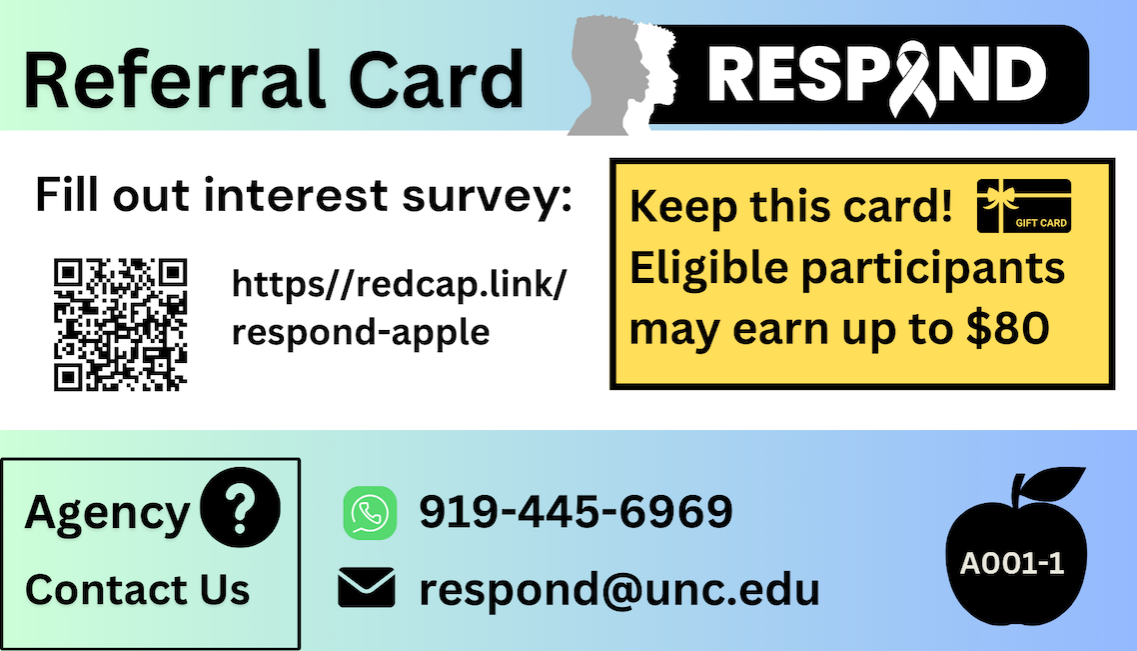
Example referral card that ambassadors distribute to peers during the enhanced social network strategy (eSNS) outreach period. Each ambassador has a unique ID and image (bottom right). The QR code and URL link to a contact form uniquely associated with each ambassador. Peers who complete the contact form have unique codes that link to the ambassador’s referral card.

During the first coaching session, ambassadors will be prompted to identify people in their social network (peers) whom they believe would benefit from linkage to HIV prevention or treatment services, including but not limited to sexual or injecting partners. Collectively, these peers comprise each ambassador’s outreach network. In addition to demographic information, ambassadors will be asked to provide each peer’s first name and last initial (or nickname or pseudonym). The DIS coach will guide the ambassador through a semistructured conversation to create a unique action plan to contact each peer (see Multimedia Appendix 1). DIS coaches will advise ambassadors that they do not need to disclose their HIV status or medical history and will help devise conversational strategies to manage situations where peers may ask about the ambassador’s HIV status. At the first coaching session, ambassadors will be supplied with printed and digital referral cards to distribute to peers in their outreach network (see Figure 6).

#### Step 3: Peer Outreach

In step 3, ambassadors will approach each peer identified in their outreach network to give them a unique referral card (associated with the ambassador’s study identifier) and encourage them to contact the eSNS team for assisted linkage to HIV services (see Figure 6). There will be no predetermined limit on the number of referral cards ambassadors can disperse, although DIS coaches will be trained to guide ambassadors to select peers whom they believe will benefit from one or more HIV services (Step 4). Peers indicate their interest by submitting a contact form to request that the eSNS team reach out to them, calling the eSNS team directly, or meeting the DIS coach in person at a time and location facilitated by the ambassador (eg, for field-based HIV testing).

We anticipate that ambassadors will engage in peer outreach for approximately 2‐6 weeks, during which DIS coaches will contact ambassadors at least weekly for brief follow-up coaching to encourage them to complete their peer outreach plan or to discuss strategies to adapt it. During follow-up coaching, new peers may be identified by the ambassador. Peer outreach will conclude when an ambassador has saturated all potential peers in their outreach network or 6 weeks have elapsed since the first coaching session, whichever comes first. Our referral period of 2‐6 weeks is a practical application of ongoing coaching and booster sessions described in implementation guidance, as SNS is based on short-term coaching [44]. Grounds for dismissal earlier than the 6-week cut-off include coercing a peer’s participation, ambassadors and peers knowingly misrepresenting their identities to obtain gift cards, repeatedly referring people not part of their network or who are ineligible, or unreliable communication following enrollment.

#### Step 4: Linking Peers to HIV Services

After a peer initiates contact with the study team, the DIS coach will attempt to follow up with that peer within 24 hours for a study screening visit. The purpose of the screening visit is to explain the study’s aims and for the DIS coach to verify whether a peer is eligible for linkage to HIV services. DIS coaches will first ask peers for their ambassador’s name to validate against the code and image on that ambassador’s referral card. Ambassadors will be compensated with a US $20 gift card per peer in their outreach network who completes screening, even if they do not enroll in the study intervention. Ambassadors will only receive compensation for a maximum of 5 peers’ screening visits (US $100 total in gift cards), although more than 5 peers may be referred.

Peers will be offered assisted linkage to HIV prevention and care services available in the Charlotte region. First, informed consent and Health Insurance Portability and Accountability Act (HIPAA) authorization will be obtained from Peers to permit the eSNS team to obtain data regarding the outcomes of scheduled HIV service appointments at community-based sites. Before scheduling appointments, DIS coaches will assess peers’ preferences among local care and prevention service providers and whether they prefer to be accompanied by their ambassador. If peers prefer, DIS coaches may also meet peers at a private location for field-based HIV testing or accompany them to a testing site. For peers who do not present with clinical symptoms of acute HIV infection, DIS coaches may perform rapid HIV testing (OraQuick HIV or bioLytical INSTI HIV dual testing) according to North Carolina DHHS protocols. Peers who test positive or indeterminate following field-based rapid testing will receive counseling by DIS coaches and expedited linkage to HIV care and bridge counseling (see the Ethical Considerations section for more detail on DIS training). Peers who reside in South Carolina and cannot attend appointments in the Charlotte region will be linked to HIV services available in Columbia, South Carolina but will not be eligible to become ambassadors.

For HIV testing, peers will be offered testing if they have never previously tested or if their last HIV test occurred more than three months prior and yielded negative or unknown results. Peers living with HIV will be linked to initial care if they are newly diagnosed or never entered care; or re-engaged with care if previously diagnosed but not currently engaged in care. Care engagement is defined as both having had a clinic visit within 12 months and currently taking ART. Peers will be offered facilitated linkage to PrEP if HIV test results obtained within the prior three months were confirmed non-reactive and they have never been on PrEP, or if they previously took PrEP but have not received a PrEP prescription in the past 6 months.

Peers will receive US $20 gift cards for attending each of up to 2 HIV service appointments, US $40 total in gift cards (eg, completing both HIV testing and attending a PrEP initiation appointment). The study team will obtain and document all HIV test results. For peers choosing to engage in HIV services at a community site unaccompanied by a DIS coach, the eSNS team will contact the site and provide the HIPAA authorization to confirm that the peer attended the appointment and, as applicable, to obtain their HIV test results. Peers may also choose to self-disclose their attendance or results to the eSNS team with a photo or screenshot. Following linkage, peers may be invited to enroll as community ambassadors and initiate the intervention cycle again by participating in coaching (Step 2) and peer outreach (Step 3).

### Ethical Considerations

The study was approved by the University of North Carolina at Chapel Hill Institutional Review Board (IRB #22‐1988). Intervention procedures were determined to present minimal risks to participants, which include potential loss of confidentiality or emotional distress caused by the potentially sensitive nature of discussing HIV or receiving an HIV diagnosis following testing facilitated by the study. Ambassadors and enrolled peer participants will provide informed consent. All DIS credentialed in North Carolina, including DIS coaches, are trained to address psychological consequences resulting from an HIV or STI diagnosis, which includes specific modules on linking people to services (medical, behavior, and social support), in-person training, and ongoing mentorship with senior DIS. The eSNS team will follow applicable North Carolina DHHS and IRB procedures for lawful reporting of safety concerns, logging unanticipated events involving study participants, and referrals to social services agencies.

Research that leverages peer relationships can create ethical dilemmas around how to minimize the risk of coercion among peers and protect the confidentiality of participants who may know each other [46]. Creating a peer outreach plan will require asking for information about individuals who are not yet enrolled—and may never enroll—in the study intervention. To avoid a scenario where identifiable information is indirectly collected about unconsented peers (making them “secondary subjects”), only first names and last initials or pseudonyms will be recorded during coaching. Despite this precaution, peers contacted by ambassadors may have unpredictable reactions to receiving outreach or discussing HIV services, including assuming that sensitive or identifiable information was provided about them. However, as no personal identifiers will be recorded unless peers initiate screening, the study team will have no way of contacting (or identifying) peers until peers contact the study team. Once peers engage in screening, DIS coaches will explain that no data collected by the study team will be shared with the ambassador who referred them.

Compensation for peer outreach was structured to minimize the possibility of creating a financial incentive for ambassadors to coerce peers into research participation. Ambassador gift cards for peer referrals are limited in both number (maximum 5) and amount (US $20). Providing ambassadors gift cards for one of their peers completing a screening visit, rather than for enrolling in the study or completing an HIV service appointment, ensures that ambassadors are fairly compensated for time spent recruiting peers while avoiding a scenario where their compensation is contingent on peers’ enrollment or participation in the study’s intervention. The screening visit includes a description of the study and eligibility survey performed by a DIS coach, who will be trained to emphasize to peers that participation is voluntary, and peers who choose not to participate but who still wish to receive HIV services (eg, HIV testing) will be provided a referral to a community site.

### Data Sources and Management

The REDCap platform hosted at UNC Chapel Hill is a HIPAA-compliant web-based platform that will serve as a central repository for data collected by the study team, including administration of ambassador and peer e-consent forms, participant recruitment and enrollment tracking, and collection of screening, appointment results, and survey data. Public health surveillance data obtained from the North Carolina Electronic Disease Surveillance System (under a data sharing agreement with North Carolina DHHS) will be stored in a secure cloud drive and REDCap. We plan to routinely analyze HIV sequences reported to the state and associated case-level epidemiological data to facilitate timely response to clusters within NC DHHS’s Region II (Charlotte).

### Data Analysis

#### Hypothesis

Our principal hypothesis posits that implementing eSNS will increase the uptake of HIV services within the Charlotte region among members of priority networks when compared to the Raleigh region over the same period. By harnessing peer social support networks, eSNS is designed to mitigate structural barriers—including stigma, distrust, limited PrEP knowledge, and difficulties navigating complicated medication logistics—that underlie disparities in HIV prevention and treatment services uptake among members of priority networks engaged by routine partner services (and among our key population). Compared to partner services alone, which is the standard of care intervention in North Carolina and South Carolina, we also hypothesize that enhancing standard SNS with genetic cluster analysis will enable further penetration into priority networks where onward HIV transmission is occurring by identifying a greater number of persons living with undiagnosed HIV that is closely related to HIV in other members of the network.

#### Effectiveness Evaluation

Intervention effectiveness will be assessed by comparing the 5-county Charlotte region to another urban North Carolina region, Raleigh, with similar demographics (see Figure 1). The metropolitan region surrounding Raleigh has the second-largest population in North Carolina, after Charlotte [47]. The primary effectiveness measures are the number of HIV-negative individuals linked to PrEP and the individuals living with HIV who are linked to care per index person from a priority network. Index persons in the intervention region (Charlotte) priority networks will be compared to index persons in the control region (Raleigh) priority networks, who receive standard-of-care partner services. Partner services are standardized among all North Carolina DHHS Field Service regions [48]. We will use a regression point displacement design (RPDD) [49] to assess the impact of eSNS on trends in our primary effectiveness measures between regions over the intervention period; the effective reproduction number for HIV will also be calculated over this period. Measures will be collected for each region at a preintervention baseline (2 years before), postintervention (6 months after), and during implementation for interim analyses at 6-month intervals. Because state HIV surveillance data is available in both the Charlotte and Raleigh regions from the baseline timepoint to a postintervention timepoint, RPDD provides statistical power to estimate the effect of eSNS when aggregating at a group level (index persons from priority networks) [50].

Although we adapted our original intervention design to enroll eSNS ambassadors through both routine partner services for index persons from priority networks and through ongoing waves of community-based recruitment, we will calculate the same effectiveness measures for ambassadors enrolled through each source. Only outcomes for ambassadors enrolled through partner services will be included in the RPDD analysis. However, for ambassadors enrolled through both sources, secondary effectiveness measures will also assess the extent to which eSNS penetrates priority networks where HIV transmission is ongoing. Among peers referred by all ambassadors, we will use a combination of study data, state HIV surveillance data, and molecular epidemiology to calculate the proportion of peers living with HIV that has close genetic linkages [42] to a cluster. We will also assess penetration into priority networks by calculating the proportion of newly diagnosed peers who initiate care, proportion of previously diagnosed peers who re-engage in care, the overall positivity among peers who test for HIV, and the proportion of peers testing negative who then initiate PrEP. Selected primary and secondary effectiveness measures are described in Table 1.

**Table 1.** Selected effectiveness measures of the enhanced social network strategy (eSNS) intervention.

Measures and description		Data source
Network Index		
Number of individuals diagnosed with HIV and linked to care per associated index case.^a^		Surveillance data
Number of HIV-negative individuals linked to PrEP^b^ per associated index case.^a^		Surveillance data
Cluster analysis		
Proportion and number of peers living with HIV who are associated with a genetic cluster involving a new HIV diagnosis.		Study and surveillance data
Peers identified with high network bridging scores (betweenness centrality) [51].		Study and surveillance data
Viral suppression		
Number of peers per ambassador previously diagnosed with HIV who are not virally suppressed.		Study and surveillance data
Care and prevention linkage		
Proportion and number of peers newly diagnosed with HIV who initiate care.		Study data
Proportion and number of peers previously diagnosed with HIV who re-engage in care.		Study data
Proportion and number of peers who test negative and initiate PrEP.		Study data
Proportion of positive tests among all peers who test for HIV.		Study data

a Primary effectiveness measures.

b PrEP: pre-exposure prophylaxis.

#### Sample Size and Power Estimates

Of approximately 280 new HIV diagnoses reported annually in the Charlotte region, 80 would meet criteria for index persons eligible to be assigned to DIS coaches for routine partner services (including 20 acute HIV infection). From these 80 index persons, we estimate that 25% of contact networks reached through partner services will yield an enrolled ambassador (n=20). Based on partner services data previously reported to North Carolina DHHS, 100 contacts were elicited from 60 persons who were newly diagnosed with HIV in the Charlotte region from 2016 to 2019, yielding a network index of 1.7 (SD 1.8). With a peer outreach network index of 5.0 per ambassador (based on previous reports [22]), this would increase the overall network index in the Charlotte region from 1.7 to 3.0. We estimate an 80% power with alpha=.05 to detect a significant effect of eSNS on network index with 20 ambassadors enrolled per year for 3 years (n=60). Of note, we do expect that ambassadors’ referral networks will have a bimodal distribution—with most having a low number of peers while a few will yield a high number of peers.

#### Process Evaluation

Process measures will be routinely collected during phase 2 to examine implementation facilitators and barriers concurrent to intervention delivery, which will inform future scale-up (see Figure 3). We will use a triangulation protocol to integrate mixed-methods data from qualitative and quantitative sources [52]. Guided by the CFIR [38], we plan to conduct in-depth interviews with 2 ambassadors per intervention year (total of 6 interviews with ambassadors) and annual interviews pre- and postimplementation with the 2 DIS coaches (total of 4 interviews). Interviews will explore DIS coach and ambassador perceptions across three CFIR domains: including (1) the adaptability and complexity of the intervention (intervention characteristics); (2) the structural characteristics, communications, relative priority of eSNS within a state public health agency delivering partner services (inner setting*)*; and (3) the characteristics of DIS coaches’ and ambassadors’ self-efficacy and knowledge (individuals). To understand additional factors that may influence intervention delivery, we developed a survey instrument administered to ambassadors after concluding peer outreach. This instrument will incorporate items from previously validated scales to quantitatively assess self-efficacy and intervention acceptability [53], HIV-related stigma (for ambassadors living with HIV) [54,55], experiences of discrimination [56], and gender identity and connectedness with the LGBTQ+ community [57,58].

Routine program monitoring data will be analyzed at 6-month intervals to assess whether eSNS is being delivered as intended (fidelity) over the intervention period and the extent to which eSNS as designed mitigates social barriers to HIV services engagement. We will monitor program delivery along each step in eSNS. Fidelity measures include the number of potential ambassadors identified per year (step 1), the length of the initial coaching session (step 2), the number of follow-up coaching contacts each DIS coach holds with each ambassador, and the length of time between peers contacting the study team and screening (step 4). A full set of monitoring measures is provided in Multimedia Appendix 2.

## Results

The study was funded in July 2022. The initial protocol for phase 1 was approved by the IRB in October 2022, and a modification detailing eSNS intervention procedures for Phase 2 was approved in June 2024. As of December 2024, we have completed the initial phase 1 formative assessments. We launched participant recruitment for eSNS in March 2025. Baseline analyses of HIV molecular epidemiology across North Carolina and South Carolina remain pending execution of data sharing agreements in both states.

Our preliminary research findings and guidance from our CAG and phase 1 identified several recommendations for adaptation and implementation of eSNS. An overarching theme in our qualitative data was the importance of fostering trust through partnerships with CBOs. This led us to develop procedures for supplementing participant recruitment of potential ambassadors beyond partner services clients in priority networks (eSNS step 1) and for facilitating HIV services linkage at local CBOs and clinics (eSNS step 4). Formative qualitative analyses also identified recommendations to address the experience of our key populations with research engagement—specifically by considering strategies to alleviate research fatigue and to ensure that our key populations are represented among the study team. Our CAG recommended opening study participation to members of the Latin community, which is increasingly affected by HIV in the Charlotte area. The amount of overlap between Black and Spanish-speaking Latin sexual networks is currently unclear. To address this, we began offering the PNS in Spanish during phase 1 and expanded the study’s key population to include both Latin and Black GBMSM and transgender women. In phase 2, English-speaking Latin persons will be included as ambassadors. Future intervention scale-up will be needed to adapt eSNS to Spanish-speaking Latine persons.

From interviews with members of our key population who self-identified as Black or African American, we found that privacy and flexibility in HIV prevention and care services were important themes. We have adopted recommendations to allow appointment scheduling for peers at nontraditional times, having DIS coaches offer peers discreet, field-based HIV rapid testing, ensuring time in between appointments to avoid peers crossing paths, and allowing ambassadors to choose their preferred mode of coaching (in-person or phone call). Finally, confusion arose about intervention roles (DIS coaches vs ambassadors) during qualitative data collection with both HIV services providers and key population members. We will train DIS coaches to clearly communicate the structure of the compensation process, and that peers may choose whether to be accompanied by their ambassador to service appointments, and that under no circumstances will ambassadors be able to access their peers’ protected health information or study data.

## Discussion

### Implications

Our study aims to provide critical information guiding “next-generation” partner services in high HIV burden areas of the Southern US that extend across urban-rural and state boundaries, leveraging active involvement from community partners impacted by HIV. Our initial intervention design incorporated several key adaptations to CDC’s SNS procedures. Compared to previous SNS pilot studies [18,20-22], eSNS expands the scope of service engagement to include PrEP and HIV care in addition to HIV testing. Importantly, we aim to specifically engage priority networks experiencing recent HIV transmission, not only demographic groups disproportionately impacted by HIV. We will focus engagement with these networks by integrating eSNS into existing health department–delivered partner services and, to assess the penetration of eSNS into priority networks, propose detailed characterization of ambassadors’ network relationships. These analyses will inform continued monitoring and adaptation of the eSNS intervention.

### Challenges and Limitations

Our rationale for understanding and leveraging recent HIV transmission networks is based on rigorous previous research [59]. We anticipated several challenges and have incorporated strategies informed by our formative work to mitigate potential problems. Challenges to recruitment could limit study success, which may be heightened within partner services, where participants may be unreachable, unwilling or unable to refer sexual partners, or distrustful of public health. We have used several strategies to mitigate this: routine elicitation of social contacts during partner services, expansion of ambassador recruitment to community sites, and robust engagement of our CAG (the latter 2 were identified by our formative research findings). Ambassadors may not have well-connected networks and may still have difficulty reaching marginalized social communities. We plan to review the composition of Ambassadors’ outreach networks on an ongoing basis, which will specifically try to identify communities that are and are not reached. In addition, potential problems can arise during eSNS implementation, such as misinterpretation of messaging around networks, fraudulent activity, and privacy violations. Informed by phase 1 formative work, we established robust community partnerships to optimize trust and foster active input in study implementation. Finally, surveillance data acquisition and management of multiple, dynamic datasets are obstacles for timely study initiation and cluster identification and require careful data management, security measures, and staff training. Obtaining surveillance data across several jurisdictions has stalled the molecular epidemiology components of our study due to legal restrictions on data sharing. As of January 2025, a data use agreement with the South Carolina Department of Public Health remains pending; lack of South Carolina surveillance data will limit identification of HIV clusters that span both states.

### Conclusions

We plan to implement an evidence-based social network strategy to increase engagement in HIV services among key populations disproportionately affected by HIV. This paper provides the protocol for the Carolinas RESPOND eSNS intervention, detailing the 3 phases of planned study design, implementation, and evaluation. Our mixed methods approach and active CAG involvement before and during implementation to adapt recruitment strategies will facilitate understanding of barriers and facilitators of peer recruitment success and allow us to make community-appropriate adaptations in real time. eSNS implementation began in March 2025, with final results expected in 2028.

## Supplementary material

10.2196/69495 Multimedia Appendix 1 Coaching guide embedded within Network Canvas software and used to help ambassadors develop a peer outreach action plan.

10.2196/69495 Multimedia Appendix 2 Program monitoring measures that will be routinely collected and analyzed to assess program performance, organized by the 4 “steps” of eSNS that they assess.

## Abbreviations

ART: antiretroviral therapy
CAG: community advisory group
CBO: community-based organization
CDC: Centers for Disease Control and Prevention
CFIR: Consolidated Framework for Implementation Research
DHHS: Department of Health and Human Services
DIS: disease intervention specialist
EHE: Ending the HIV Epidemic
eSNS: enhanced social network strategy
GBMSM: gay, bisexual, and other cisgender men who have sex with men
HIPAA: Health Insurance Portability and Accountability Act
MSEM: modified social ecological model
PrEP: pre-exposure prophylaxis
REDCap: Research Electronic Data Capture
RPDD: regression point displacement design
SNS: social network strategy
