# Supplementary material for: An Enhanced Social Network Strategy to Increase the Uptake of HIV Services: Protocol for Type I Hybrid Implementation Study (Carolinas RESPOND)
Source: JMIR Public Health Surveill. 2025 Aug 29;11:e69495. doi: 10.2196/69495 (PMC12396802; doi:10.2196/69495)
Supplement: Multimedia Appendix 1 [file publichealth-v11-e69495-s001.pdf]

IGHID12218_v4_Coaching Guide.netcanvas

This Network Canvas tool will help eSNS Ambassadors and Coaches to identify potential people to refer and help develop a plan for contacting them. This version was included in an IRB modification for Phase 2 submitted in October 2024.

LAST MODIFIED:10/28/2024, 9:13:57 AM

SCHEMA VERSION:7

DOCUMENT CREATED: 10/29/2024 9:29:54 AM

Contents

STAGES

1. Introduction
2. Ambassador Info
3. Coaching Session Information
4. Check Understanding
5. Referral Card
6. Compensation Introduction
7. Compensation & Other Questions
8. Confidentiality
9. Introducing Naming
10. Identify Peers to Link
11. Ambassador's Relationship with Peers
12. Comfort Discussing
13. HIV Status
14. PLWH Follow-Up
15. PNLWH Follow-Up
16. Role-Playing Outreach
17. Services
18. Reason to Link Peer
19. Peer's Test Result
20. Peer Barriers
21. Planning Peer Outreach
22. Another Contact Method
23. Remove Peer
24. Referral Map
25. Coach Follow-Up Planning
26. Ambassador Demographics
27. Peer Demographics
28. Peer-to-Peer Relationship Strength
29. Wrapping Up the Plan
30. Final Ambassador Questions

CODEBOOK

Ego

NODE TYPES

Peer

EDGE TYPES

alter_closeness

ASSETS

IMAGE

- Charlotte_Mecklenburg.png
- RESPOND Logo.png
- CLT PrEP Map.png
- Incentives Table.png
- Screenshot 2024-05-24 at 2.59.33 PM.png
- Screenshot 2024-08-12 at 9.42.40 AM.png
- Screenshot 2024-10-08 at 1.50.36 PM.png
- Incentives.png
- Incentives.png
- IGHID_12218_Peer_Referral_Card_v3_2024-10-09.png
- RESPOND Logo [2024].png
- Screenshot 2024-10-28 at 9.13.25 AM.png
- Testing Map.png

1 Introduction

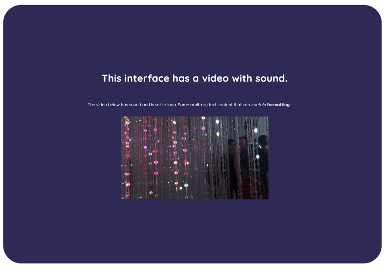

PAGE HEADING

Reaching Out to Your Peers for the Social Network Strategy (SNS)

ITEMS

NAME	RESPOND Logo [2024].png
BLOCK SIZE	LARGE
TYPE	Image
PREVIEW	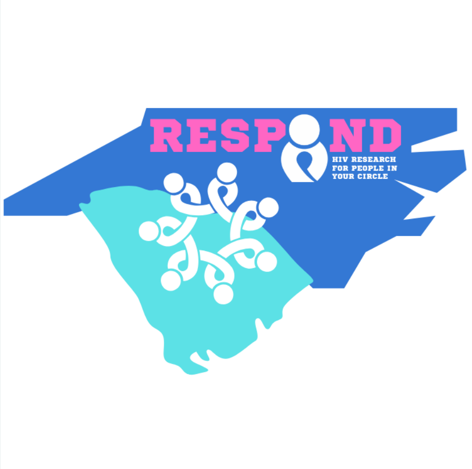

INTERVIEWER SCRIPT

2 Ambassador Info

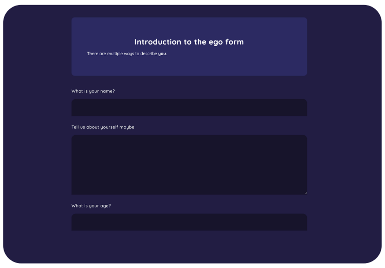

INTRODUCTION PANEL

Your Info

What name would you like to be called?

FORM

VARIABLE	COMPONENT	PROMPT
nickname	Text	What name do you go by?
name	Text	Full Name
phone	Number	What's a good phone number for you?

INTERVIEWER SCRIPT

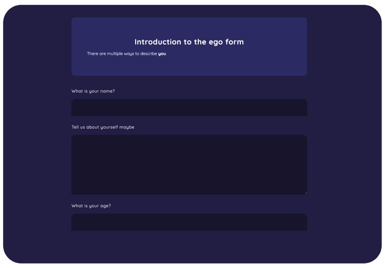

3 Coaching Session Information

INTRODUCTION PANEL

Coaching Session #1

Today's Coaching Session

FORM

VARIABLE	COMPONENT	PROMPT
coaching1_date	RelativeDatePicker	Today's Date
amb_id	Text	Re-enter the Ambassador's ID associated with their consent form in REDCap. For example, A-1001.
coach	RadioGroup	Coach Name

INTERVIEWER SCRIPT

4 Check Understanding

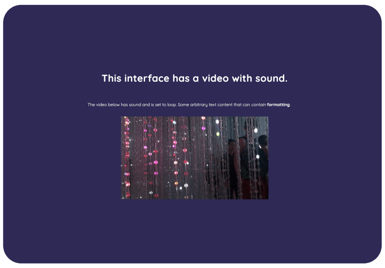

PAGE HEADING
Getting Started

ITEMS

BLOCK SIZE	MEDIUM
TYPE	Text
CONTENT	Tell me what your understanding is at this point of what you'll be doing.

BLOCK SIZE	MEDIUM
TYPE	Text
CONTENT	Is this something you're still interested in doing?

INTERVIEWER SCRIPT

What is your understanding of what I am asking you to do?

At this point, the Ambassador has completed orientation.

This question is asked because the Coach wants to assess whether the Ambassador has a clear understanding of what they are being asked to do.

If there is a clear and complete understanding, the Coach can move forward. If not, the coach should correct misunderstandings and provide vital information.

Is this still something you're interested in doing?

5 Referral Card

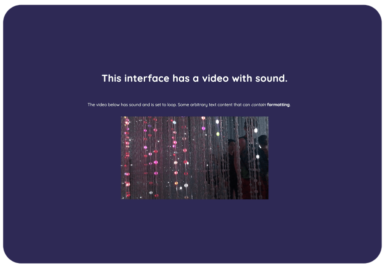

PAGE HEADING

Referral Card

ITEMS

**BLOCK SIZE**	SMALL
**TYPE**	Text
**CONTENT**	Here's an example of the referral cards you'll give to your peers.

**NAME**	IGHID_12218_Peer_Referral_Card_v3_2024-10-09.png
**BLOCK SIZE**	MEDIUM
**TYPE**	Image
**PREVIEW**	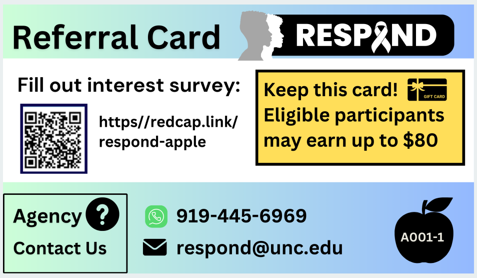

INTERVIEWER SCRIPT

6 Compensation Introduction

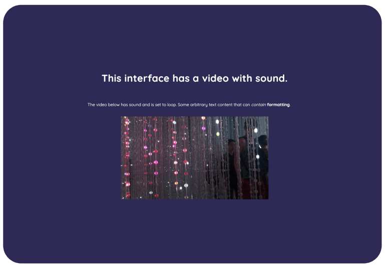

PAGE HEADING

Compensation

ITEMS

BLOCK SIZE	SMALL
TYPE	Text
CONTENT	Let’s talk about how compensation, like gift cards, work for this program.

NAME	Screenshot 2024-10-28 at 9.13.25 AM.png
BLOCK SIZE	MEDIUM
TYPE	Image
PREVIEW	

Study Activity	Ambassador	Peer
Personal Network Survey	$40	$40
Ambassador Interview	$50	---
Coaching Session #1	$35	---
Ambassador Graduation Survey	$40	---
Peer completes screening visit	$20 x 5 (Max $100)	---
Peer completes HIV test<br>Peer completes PrEP appointment<br>Peer completes HIV Care appointment	---	$20 x 2 (Max $40)

BLOCK SIZE	SMALL
TYPE	Text
CONTENT	You can reach out to more than 5 people and reach out to more people than we discuss today. But you will only be compensated for up to 5 people who complete a screening visit.

INTERVIEWER SCRIPT

7 Compensation & Other Questions

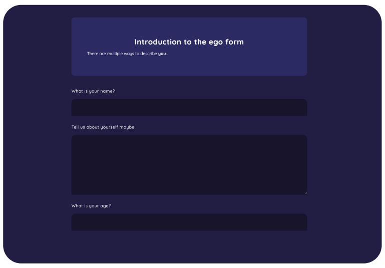

INTRODUCTION PANEL

Compensation

Let's talk about any questions you have about compensation or participating.

FORM

VARIABLE	COMPONENT	PROMPT
incentives_questions	TextArea	What questions do you have?
incentives_explain	TextArea	How would you explain this to people you're recruiting?

INTERVIEWER SCRIPT

8 Confidentiality

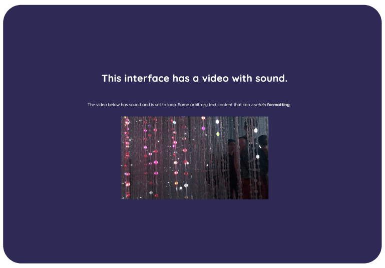

PAGE HEADING

Confidentiality

ITEMS

**BLOCK SIZE**	MEDIUM
**TYPE**	Text
**CONTENT**	Let’s talk about confidentiality. None of the information you have provided me with will be shared with anyone outside our study team. The information we are collecting on the tablet will be kept confidentially and used as a reminder of how you plan to reach out each person.

INTERVIEWER SCRIPT

The Coach should explain study procedures for data security using simple language.

9 Introducing Naming

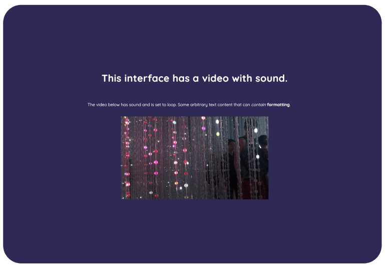

PAGE HEADING

Your Peers

ITEMS

**BLOCK SIZE**	MEDIUM
**TYPE**	Text
**CONTENT**	Next, I'm going to ask you to think of people you know who may benefit from linking to HIV or sexual health services.<br><br>Some other terms for people like this are "peers" or "people in your circle".<br><br>I'll ask you for their first name and last initial.

INTERVIEWER SCRIPT

10 Identify Peers to Link

SUBJECT	Peer
VARIABLES	name

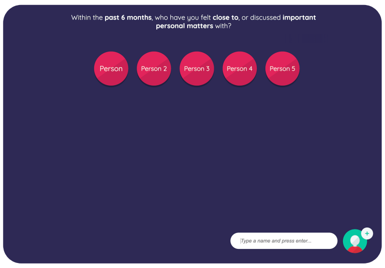

QUICK ADD

VARIABLE	name
TYPE	text

PROMPTS

1. Next, I'd like for us to talk about someone you know who you think may benefit from testing for HIV, or getting linked to other HIV or sexual health services, like PrEP. What would you like to call them?

BEHAVIOURS

MINIMUM NODES ON STAGE	1

INTERVIEWER SCRIPT

11 Ambassador's Relationship with Peers

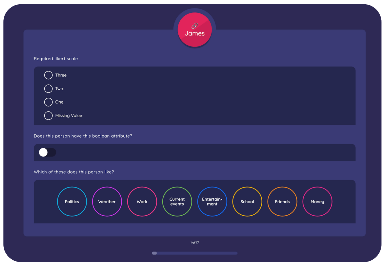

SUBJECT	Peer
VARIABLES	closeness, relationship_type

INTRODUCTION PANEL

Your Relationships

Tell me about your relationships with each person.

FORM

VARIABLE	COMPONENT	PROMPT
relationship_type	ToggleButtonGroup	Tell me about what type of relationship you have with this person.
closeness	LikertScale	How close are you to this person?

INTERVIEWER SCRIPT

12 Comfort Discussing

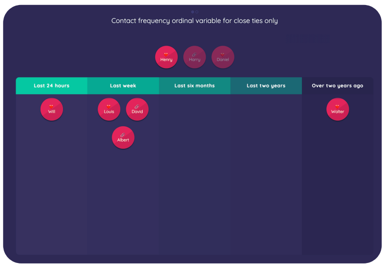

SUBJECT	Peer
VARIABLES	comfort

PROMPTS

1. How comfortable do you feel talking to each person about HIV and/or sexual health?

VARIABLE	comfort

INTERVIEWER SCRIPT

13 HIV Status

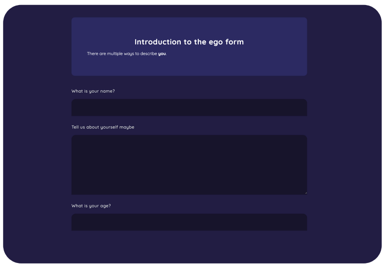

INTRODUCTION PANEL

Experiences with HIV Services

You may choose whether or not to share your experiences with HIV-related healthcare services. Let's talk about your experiences so we can plan how to approach conversations with your peers. Remember, everything we talk about is confidential.

FORM

VARIABLE	COMPONENT	PROMPT
status	RadioGroup	What is your HIV status?

INTERVIEWER SCRIPT

14 PLWH Follow-Up

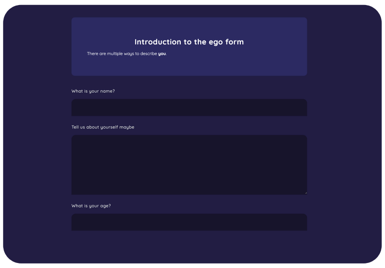

SKIP LOGIC

ACTION	SHOW
RULES	Ego has status that is exactly equal to **Living with HIV**

INTRODUCTION PANEL

Experiences with HIV Services

Tell me a bit more about your experiences being diagnosed with HIV.

FORM

VARIABLE	COMPONENT	PROMPT
diagnosis_year	DatePicker	What year were you diagnosed?
diagnosis_experiences	TextArea	What were your experiences like with HIV treatment services?

INTERVIEWER SCRIPT

15 PNLWH Follow-Up

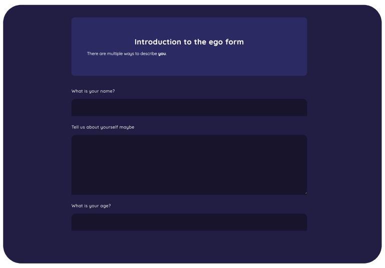

SKIP LOGIC

**ACTION**	SHOW
**RULES**	Ego has status that is exactly equal to **Negative**

INTRODUCTION PANEL

Experiences with HIV Services

Tell me a bit more about your experiences with HIV-related services like PrEP or testing.

FORM

VARIABLE	COMPONENT	PROMPT
prep	RadioGroup	Are you currently taking PrEP?
prevention_experiences	TextArea	What are your experiences like with HIV prevention services like PrEP or testing?

INTERVIEWER SCRIPT

16 Role-Playing Outreach

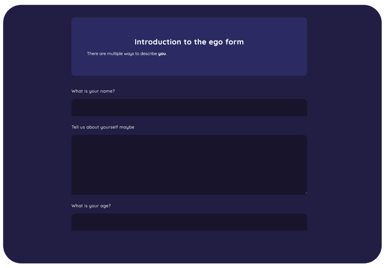

INTRODUCTION PANEL

Practicing Conversations

Let's think through some situations...

FORM

VARIABLE	COMPONENT	PROMPT
conversation	Text	How would you bring up the subject of HIV services with the people in your circle we've talked about? What will you say about it?
disclosure	Text	Do you think anyone we've talked about will ask you about your HIV status? If so, how would you respond?
disclosure_alternatives	Text	If you are not comfortable disclosing your status, whether positive or negative, how can you discuss the importance of sexual health and HIV services without revealing your HIV status?

INTERVIEWER SCRIPT

Avoid making recommendations about disclosure. Discuss approaches for disclosing HIV status if they choose to do so. Discuss approaches to raising the topic of HIV services without revealing their own status. Ask the Ambassador how they would talk about the importance of testing without revealing their HIV status.

17 Services

SUBJECT	Peer
VARIABLES	service_type

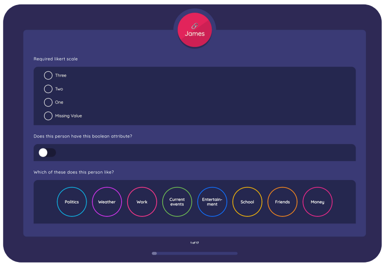

INTRODUCTION PANEL

Types of Services

This program is all about helping people you know get the sexual health services they need.

FORM

VARIABLE	COMPONENT	PROMPT
service_type	ToggleButtonGroup	What type of service do you think this person benefit from? If you're not sure, we can skip this question.

INTERVIEWER SCRIPT

18 Reason to Link Peer

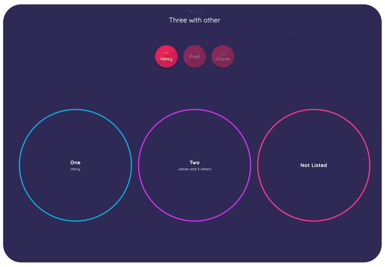

**SUBJECT**	Peer
**VARIABLES**	reason, reason_other, testing

PROMPTS

1. What is the main reason you think this person may benefit from linking to HIV or sexual health services?

**VARIABLE**	reason
**OTHER VARIABLE**	reason_other
**OTHER VARIABLE PROMPT**	What made you think of this person when we started?
**OTHER OPTION LABEL**	Another reason

2. Do you believe this person has ever tested for HIV before?

**VARIABLE**	testing

INTERVIEWER SCRIPT

In the risk assessment question, the Coach is assessing how well the Ambassador knows the Peer and whether the Ambassador has a clear understanding of which services may be appropriate. If the Ambassador does not provide any information indicating reasons the Peer may benefit from linkage, it may be necessary to review what risk behavior is and try to determine whether the Ambassador believes the Peer is at risk for transmission of or exposure to HIV.

19 Peer's Test Result

SUBJECT	Peer
VARIABLES	test_result, testing

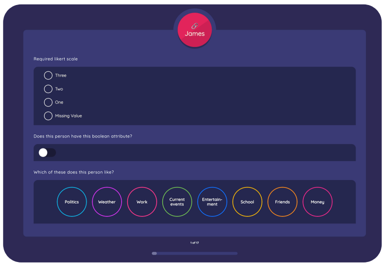

NETWORK FILTERING

RULES	Peer where testing includes Yes

SKIP LOGIC

ACTION	SHOW
RULES	Peer where testing includes Yes

INTRODUCTION PANEL

Peers

A follow-up question about testing...

FORM

VARIABLE	COMPONENT	PROMPT
test_result	RadioGroup	Do you know what the most recent HIV test result was?

INTERVIEWER SCRIPT

20 Peer Barriers

SUBJECT	Peer
VARIABLES	barriers_notes, peer_barriers

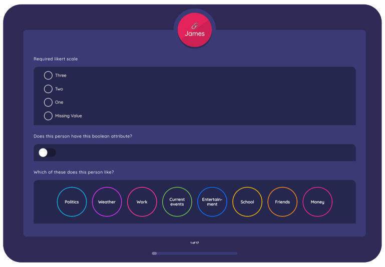

INTRODUCTION PANEL

Challenges for Peers

Now let's think about any challenges your peers might have participating.

FORM

VARIABLE	COMPONENT	PROMPT
peer_barriers	ToggleButtonGroup	What challenges, if any, do you think they might have linking to services?
barriers_notes	TextArea	Anything else that might be a challenge for this person?

INTERVIEWER SCRIPT

21 Planning Peer Outreach

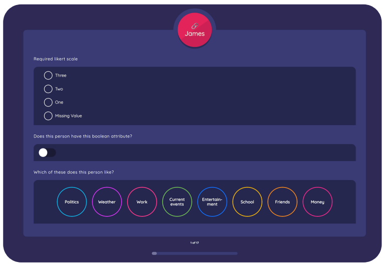

**SUBJECT**	Peer
**VARIABLES**	contact_date, contact_type, peer_rxn, referral_type

INTRODUCTION PANEL

Making a Plan

Now that we've talked about people in your circle, let's make a plan for you reaching out to them.

FORM

VARIABLE	COMPONENT	PROMPT
contact_type	ToggleButtonGroup	What would be the best way to bring up subject of HIV and/or sexual health services with this person? Choose 1 to start.
referral_type	ToggleButtonGroup	What do you think is the best way to link this person to services?
peer_rxn	RadioGroup	Do you have any concerns for your safety or this person's safety as a result of recruiting them? For example, someone becoming angry or physical.
contact_date	RelativeDatePicker	When could you reach out to them?

INTERVIEWER SCRIPT

22 Another Contact Method

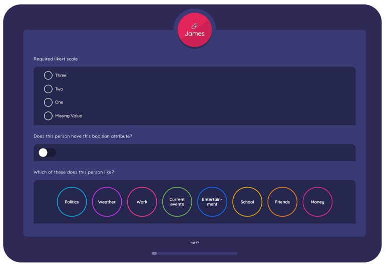

**SUBJECT**	Peer
**VARIABLES**	contact_type, contact_type_other

NETWORK FILTERING

**RULES**	Peer where contact_type includes **Another way**

SKIP LOGIC

**ACTION**	SHOW
**RULES**	Peer where contact_type includes **Another way**

INTRODUCTION PANEL

Another Contact Method

What would be the best way to reach out to this person?

FORM

VARIABLE	COMPONENT	PROMPT
contact_type_other	Text	Say a bit more about how you could get in touch...

INTERVIEWER SCRIPT

23 Remove Peer

SUBJECT	Peer
VARIABLES	concerns_pin, peer_rxn, remove_pin

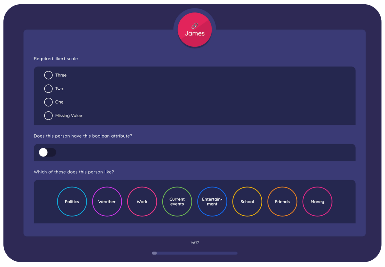

NETWORK FILTERING

RULES

Peer where peer_rxn is exactly equal to **Yes**

OR

Peer where peer_rxn is exactly equal to **Not sure**

SKIP LOGIC

ACTION	SHOW

RULES

Peer where peer_rxn is exactly equal to **Yes**

OR

Peer where peer_rxn is exactly equal to **Not sure**

INTRODUCTION PANEL

Removing People from Your Recruitment List

Let's talk about your concerns reaching out to specific people on your list.

FORM

VARIABLE	COMPONENT	PROMPT
concerns_pin	TextArea	Tell me more about your concerns.
remove_pin	RadioGroup	Should we remove this person from your list?

INTERVIEWER SCRIPT

24 Referral Map

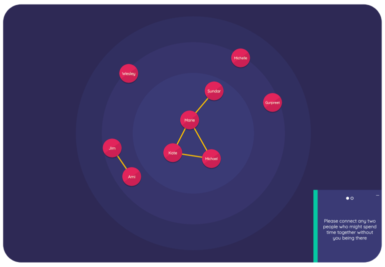

SUBJECT	Peer
VARIABLES	service_site

PROMPTS

1. Where do you think this person is most likely to come for services?

LAYOUT VARIABLE	service_site
ALLOW HIGHLIGHTING	*FALSE*

INTERVIEWER SCRIPT

25 Coach Follow-Up Planning

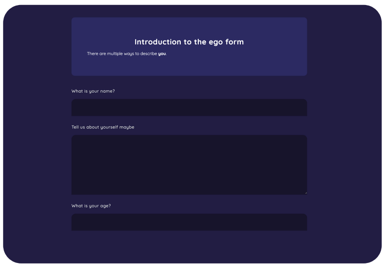

INTRODUCTION PANEL

Following Up

Let's plan our next time to talk and to see how things went with your peers.

FORM

VARIABLE	COMPONENT	PROMPT
card_method	ToggleButtonGroup	How would you like your referral cards? We can give you physical cards now, send you digital ones later, or both.
fu_contact	ToggleButtonGroup	What's the best way to check in again?
fu_frequency	LikertScale	How often should we schedule our check-ins?
fu_date	RelativeDatePicker	When is a good time to check in again?
email	Text	What's the best email address to send your compensation to?

INTERVIEWER SCRIPT

Arrange for follow-up between you and Ambassador in order to problem solve, reinforce, support and identify additional Peers. It is recommended that the follow up with the Ambassador take place as soon as possible after the Ambassador's planned conversation with each Peer.

26 Ambassador Demographics

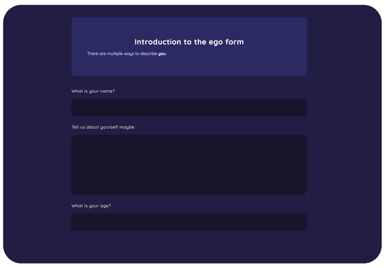

INTRODUCTION PANEL

Your Info

A few demographic questions about you to finish up...

FORM

VARIABLE	COMPONENT	PROMPT
dob	DatePicker	Date of Birth
race	ToggleButtonGroup	Race (choose all that apply)
ethnicity	RadioGroup	Ethnicity
gender	RadioGroup	How would you describe your gender?

INTERVIEWER SCRIPT

27 Peer Demographics

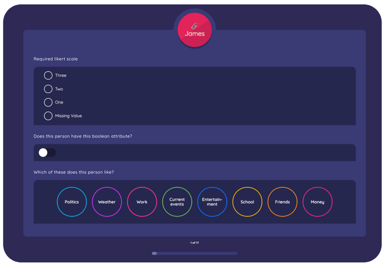

SUBJECT	Peer
VARIABLES	age_c, gender2, race

INTRODUCTION PANEL

Demographics of People in Your Circle

... And please tell a little more about the people you'll be reaching out to. If you don't know, we can skip any question.

FORM

VARIABLE	COMPONENT	PROMPT
race	ToggleButtonGroup	How would you describe their race? Choose all that apply.
gender2	RadioGroup	How would you describe their gender?
age_c	RadioGroup	What's your best guess of their age?

INTERVIEWER SCRIPT

28 Peer-to-Peer Relationship Strength

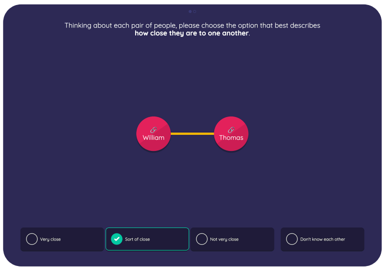

SUBJECT	Peer
VARIABLES	closeness

INTRODUCTION PANEL

Peer Relationships

Finally, tell me about how well your peers knows each other.

PROMPTS

1. How would you describe the relationship between these two people?

CREATES EDGE	alter_closeness
EDGE STRENGTH VARIABLE	closeness
NEGATIVE OPTION LABEL	Strangers

INTERVIEWER SCRIPT

29 Wrapping Up the Plan

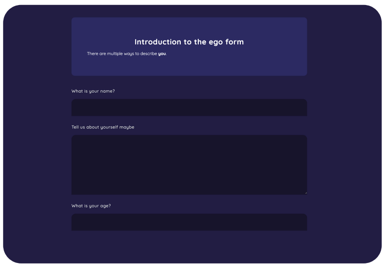

INTRODUCTION PANEL

Wrapping Up the Plan

Let's wrap up today's session...

FORM

VARIABLE	COMPONENT	PROMPT
confidence	Text	How confident do you feel that you can carry out this plan with your peers?

INTERVIEWER SCRIPT

30 Final Ambassador Questions

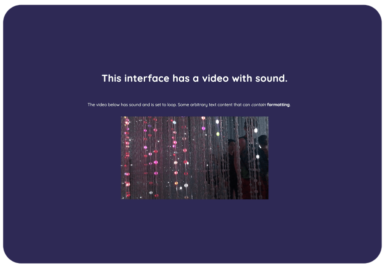

PAGE HEADING

Questions?

ITEMS

BLOCK SIZE	MEDIUM
TYPE	Text
CONTENT	What final questions or concerns do you have?

INTERVIEWER SCRIPT

Ego

Name	Type	Used In
amb_id	text	Coaching Session Information
card_method	categorical<br>VALUE / LABEL<br>1 — Digital cards<br>2 — Physical	Coach Follow-Up Planning
coach	ordinal<br>VALUE / LABEL<br>1 — Jesse<br>2 — Bryce<br>3 — Mario	Coaching Session Information
coaching1_date	datetime	Coaching Session Information
confidence	text	Wrapping Up the Plan
conversation	text	Role-Playing Outreach
diagnosis_experiences	text	PLWH Follow-Up
diagnosis_year	datetime	PLWH Follow-Up
disclosure	text	Role-Playing Outreach
disclosure_alternatives	text	Role-Playing Outreach

Name	Type	Used In
dob	datetime	Ambassador Demographics
email	text	Coach Follow-Up Planning
ethnicity	ordinal<br>VALUE / LABEL<br>1 – Hispanic/Latino/Latinx<br>0 – Not Hispanic/Latino/Latinx	Ambassador Demographics
fu_contact	categorical<br>VALUE / LABEL<br>1 – Text<br>2 – WhatsApp<br>3 – Call<br>4 – In-Person	Coach Follow-Up Planning
fu_date	datetime	Coach Follow-Up Planning
fu_frequency	ordinal<br>VALUE / LABEL<br>1 – Every day<br>2 – Every 3 days<br>3 – Every week<br>4 – Every 2 weeks	Coach Follow-Up Planning

Name	Type	Used In
gender	ordinal<br>VALUE / LABEL<br>1 Cisgender man<br>2 Cisgender woman<br>3 Transgender man<br>4 Transgender woman<br>5 Non-binary<br>99 Another gender identity	Ambassador Demographics
incentives_explain	text	Compensation & Other Questions
incentives_questions	text	Compensation & Other Questions
name	text	Ambassador Info
nickname	text	Ambassador Info
phone	number	Ambassador Info
prep	ordinal<br>VALUE / LABEL<br>1 Yes<br>2 No<br>3 Took PrEP in the past but not currently	PNLWH Follow-Up
prevention_experiences	text	PNLWH Follow-Up

Name	Type	Used In
race	categorical	Ambassador Demographics
status	ordinal	HIV Status<br>PLWH Follow-Up<br>PNLWH Follow-Up

race (categorical):

VALUE	LABEL
1	Black or African American
2	White
3	American Indian/Alaska Native
4	Asian
5	Native Hawaiian or Other Pacific Islander

status (ordinal):

VALUE	LABEL
1	Living with HIV
0	Negative
99	Prefer not to answer

Peer

Name	Type	Used In
age_c	ordinal<br><br>VALUE / LABEL<br>1 — 18-24<br>2 — 25-29<br>3 — 30-34<br>4 — 35-39<br>5 — 40-44<br>6 — 45-49<br>7 — 50-54<br>8 — 55-59<br>9 — 60 or older	Peer Demographics
barriers_notes	text	Peer Barriers
closeness	ordinal<br><br>VALUE / LABEL<br>1 — Not very close<br>2 — In-between<br>3 — Very close	Ambassador's Relationship with Peers
comfort	ordinal<br><br>VALUE / LABEL<br>4 — Very comfortable<br>3 — Somewhat comfortable<br>1 — A little comfortable<br>0 — Not at all comfortable	Comfort Discussing

Name	Type	Used In
concerns_pin	text	Remove Peer
contact_date	datetime	Planning Peer Outreach
contact_type	categorical<br><br>**VALUE** / **LABEL**<br>1 / Over text<br>2 / During an in-person conversation<br>3 / DM on social media<br>4 / Phone call<br>99 / Another way	Planning Peer Outreach<br>Another Contact Method<br>Another Contact Method
contact_type_other	text	Another Contact Method
gender2	ordinal<br><br>**VALUE** / **LABEL**<br>1 / Cisgender man<br>2 / Cisgender woman<br>3 / Transgender man<br>4 / Transgender woman<br>5 / Non-binary<br>99 / Another gender identity	Peer Demographics
name	text	Identify Peers to Link

Name	Type	Used In
peer_barriers	categorical<br><br>VALUE / LABEL<br>1 Scheduling<br>2 Transportation<br>3 Communication<br>4 Stigma<br>5 Finances<br>6 Housing	Peer Barriers
peer_rxn	ordinal<br><br>VALUE / LABEL<br>1 Yes<br>0 No<br>99 Not sure	Planning Peer Outreach<br>Remove Peer<br>Remove Peer<br>Remove Peer<br>Remove Peer
race	categorical<br><br>VALUE / LABEL<br>1 Black or African American<br>2 White<br>3 American Indian/Alaska Native<br>4 Asian<br>5 Native Hawaiian or Other Pacific Islander	Peer Demographics

Name	Type	Used In	VALUE	LABEL
reason	categorical	Reason to Link Peer	1	Has never tested for HIV
			2	Has more than 1 sex partner
			3	Has a history of STIs
			4	Has engaged in sex work
			5	Has had condomless sex without PrEP
reason_other	text	Reason to Link Peer		
referral_type	categorical	Planning Peer Outreach	1	Giving them information to follow up with a Coach
			2	Me going with them to an appointment
			3	Me coordinating with a Coach to schedule an appointment for them

Name	Type	Used In
relationship_type	categorical	Ambassador's Relationship with Peers
remove_pin	ordinal	Remove Peer
service_site	layout	Referral Map
service_type	categorical	Services
test_result	ordinal	Peer's Test Result

relationship_type (categorical):

VALUE	LABEL
1	Partner
7	Casual Partner
2	Family
3	Friend
4	Advisor
5	Co-Worker
6	Acquaintance
99	Other

remove_pin (ordinal):

VALUE	LABEL
1	Yes
0	No

service_type (categorical):

VALUE	LABEL
1	HIV test
2	PrEP
3	HIV care

test_result (ordinal):

VALUE	LABEL
1	Positive
0	Negative
99	Don't know

Name	Type	Used In
testing	categorical<br><br>VALUE / LABEL<br>0 / No<br>1 / Yes<br>99 / Don't know	Reason to Link Peer<br>Peer's Test Result<br>Peer's Test Result

alter_closeness

Name	Type	Used In
closeness	ordinal	Peer-to-Peer Relationship Strength

VALUE	LABEL
1	Not very close
2	In-between
3	Very close
99	I don't know
