# Supplementary material for: An Enhanced Social Network Strategy to Increase the Uptake of HIV Services: Protocol for Type I Hybrid Implementation Study (Carolinas RESPOND)
Source: JMIR Public Health Surveill. 2025 Aug 29;11:e69495. doi: 10.2196/69495 (PMC12396802; doi:10.2196/69495)
Supplement: Multimedia Appendix 2 [file publichealth-v11-e69495-s002.docx]

# S2. Table of eSNS Process and Monitoring Measures

| **eSNS Step** | **Indicator** |
| --- | --- |
| **1.** Finding Ambassadors | Referral source for potential eSNS Ambassadors |
|  | Proportion of Ambassadors who report they are "familiar" or "somewhat familiar" with local HIV services |
|  | Number of orientation sessions per potential Ambassador |
|  | Number of eligible Ambassadors screened but not enrolled |
|  | Proportion of eligible Ambassadors who proceed to orientation |
|  | Number of potential Ambassadors screened |
|  | Number of potential Ambassadors screened who do not meet eligibility criteria |
|  | Number of potential Ambassadors oriented |
|  | Number of Ambassadors enrolled per year |
|  | Proportion of Ambassadors enrolled from eligible index networks |
|  | Number of days between potential Ambassador entering screener data and complete orientation |
| **2.** Ambassador Coaching | Number of minutes of initial coaching session |
|  | Time from initial coaching session to first follow-up |
|  | Proportion of Peers identified by Ambassador in planned Peer outreach who are also in the priority population |
|  | Number of Peers identified per Ambassador in planned Peer outreach |
| **3.** Peer Outreach | Number of follow-up coaching contacts per Ambassador |
|  | Proportions of different types of support Ambassador plans for Peers |
|  | Median number of days between Ambassador naming Peers and Peers being screened |
| **4.** Linking Peers to HIV Services | Proportion of Ambassadors’ Peers who enroll in linkage to services through eSNS |
|  | Proportion of Ambassadors’ Peers who complete linkage to at least 1 service through eSNS |
|  | Proportion of Ambassadors’ Peers who complete linkage to all planned services through eSNS |
|  | Median number of days between Ambassador naming Peers and Ambassadors' Peers linkage appointments being scheduled |
|  | Number of hours between Peer entering contact information and study team's first follow-up contact |
|  | Number of hours between Peer entering contact information and screening |
|  | Number of days between Peer entering contact information and consenting |
|  | Number of days between Peer entering contact information and first appointment |
|  | Proportion of Peers who enroll in linkage who are also in the key population |
|  | Proportions of types of appointments sought by Peers |
|  | Proportion of Peers who receive HIV services through the study team |
|  | Proportion of Peers with HIV associated with a cluster |
|  | Peers identified with high network bridging scores |
